# Differential Impact of Plasma Homocysteine Levels on the Periventricular and Subcortical White Matter Hyperintensities on the Brain

**DOI:** 10.3389/fneur.2019.01174

**Published:** 2019-11-07

**Authors:** Kee Ook Lee, Min-Hee Woo, Darda Chung, Jung-Won Choi, Nam-Keun Kim, Ok-Joon Kim, Seung-Hun Oh

**Affiliations:** ^1^Department of Neurology, CHA Bundang Medical Center, Cha University, Seongnam-si, South Korea; ^2^Institute for Clinical Research, CHA Bundang Medical Center, Cha University, Seongnam-si, South Korea

**Keywords:** white matter hyperintensities, homocysteine, periventricular white matter, deep subcortical white matter, Fazekas score

## Abstract

**Background:** The clinical significance of cerebral white matter hyperintensities (WMH) on brain magnetic resonance imaging (MRI) has recently increased, and recognized now as a risk factor for future stroke and dementia. High levels of plasma homocysteine (Hcyt) are associated with cerebral WMH. Recent studies suggest a different anatomy and physiology in the arteriolar system may be supplied to the periventricular and deep subcortical white matter. We hypothesize that plasma Hcyt levels have differing impacts on periventricular WMH (PVWMH) than on deep subcortical WMH (DSWMH).

**Methods:** We evaluated plasma Hcyt levels from 937 neurologically healthy participants. The severity of PVWMH and DSWMH was evaluated by the use of a manual grading scale. Moderate to severe PVWMH and DSWMH levels were defined when the Fazekas score was two or three, respectively. Predominant PVWMH (pred-PVWMH) and predominant DSWMH (pred-DSWMH) were defined as having a difference of Fazekas score between PVWMH and DSWMH of two or more. Other confounding variables including age, sex, vascular risk factors, and estimated glomerular filtration rate (eGFR) were also analyzed.

**Results:** Logistic regression revealed that, after adjusting for the confounding variables, PVWMH was associated with old age, hypertension, diabetes mellitus, low eGFR, and high plasma Hcyt levels. DSWMH was associated with old age, hypertension, and hypercholesterolemia but not with plasma Hcyt levels. Plasma Hcyt levels were associated with pred-PVWMH but not with pred-DSWMH.

**Conclusions:** High plasma Hcyt levels are strongly associated with the development of PVWMH but not DSWMH. Our results suggest the possibility that different pathogeneses exist for PVWMH and DSWMH and that dysregulated Hcyt metabolism associated with the development of PVWMH.

## Introduction

Cerebral white matter hyperintensities (WMH) are visualized as hyperintense signals scattered on cerebral white matters in T2-weighted or fluid attenuation inversion recovery (FLAIR) images of brain magnetic resonance imaging (MRI) (1). Although it may be asymptomatic, several clinical studies have explored the relationship between the progression of cerebral WMH and the occurrence of various neurological diseases, such as future stroke, post-stroke depression, post-stroke cognitive decline, aging, and dementia (2–6). Despite its high prevalence and pathological substrate for various neurological diseases, the pathomechanism of cerebral WMH is not fully elucidated. The known risk factors for cerebral WMH are aging and hypertension, and less frequently, diabetes, and metabolic syndrome (7–9). Recent emerging evidence proposes endothelial dysfunction as a novel pathomechanism of cerebral WMH (10–13). As homocysteine (Hcyt) is a potential disruptor for endothelial cell function, it is plausibly a pathogen for cerebral WMH. Indeed, several clinical observations have demonstrated that elevated plasma Hcyt levels contribute to the development of cerebral small vessel diseases such as lacune and cerebral WMH (14–17). In particular, a recent Mendelian randomization study explored the association of genetically determined homocysteine levels with risk of stroke subtypes and found associations with small vessel strokes, thus suggesting a specific effect on small vessel disease (18).

Cerebral WMH is commonly categorized into two types, depending on lesional location, either periventricular WMH (PVWMH) or deep subcortical WMH (DSWMH) (19). These two WMH types usually develop and progress concurrently. However, an increasing number of studies have demonstrated that these two WMH types have different anatomical and histopathologic findings, suggesting different mechanistic pathogeneses between them (20–23). There are few studies investigating the differential impact of plasma Hcyt levels between PVWMH and DSWMH (15, 20, 24, 25). The aim of this study, therefore, is to investigate whether plasma Hcyt levels are differentially associated with PVWMH and DSWMH in a neurologically healthy population.

## Patients and Methods

### Study Population

Neurologically healthy participants 40 years or older were recruited from the outpatient clinic of the Department of Neurology at CHA Bundang Medical Center for a scheduled health examination between March 2008 and December 2010. The study was conducted according to the guidelines of the Declaration of Helsinki and approved by the Institutional Ethical Committee of the CHA Bundang Medical Center (IRB approval no. 2010-083). A retrospective analysis was designed for subjects who arrived for medical attention because they had underlying cardiovascular risk factors or a family history of stroke. Medical records, laboratory results, and radiological findings were reviewed in all subjects. Only participants whose records contained adequate information on demographic, laboratory, and radiological data were included. Of the 1,059 study subjects, 122 were excluded for various reasons, including for inadequate clinical information (*n* = 109) and the presence of a suspected neurological disease in their medical history (*n* = 13). Therefore, a total of 937 subjects were included in this study.

### Risk Factor Assessment

We reviewed patients' medical records in order to gather information on their medical history and laboratory data related to cardiovascular risk factors. Hypertension was defined as having a high baseline blood pressure (systolic ≥140 mm Hg or diastolic ≥90 mm Hg) or a history of antihypertensive treatment. Diabetes mellitus was defined as having fasting plasma glucose of ≥126 mg/dL or a history of hypoglycemic therapy. Smoking was defined as current smoker at the time of examination. Hypercholesterolemia was defined as fasting serum total cholesterol of ≥220 mg/dL or a history of taking statin medication. Data of coronary arterial occlusive disease (CAOD) was defined as presence of a history of CAOD and percutaneous coronary interventions or coronary artery bypass grafting. In addition to previously mentioned clinical data, systolic and diastolic blood pressure, serum total cholesterol, triglycerides (TG), fasting glucose, and estimated glomerular filtration rate (eGFR) were also measured.

Plasma Hcyt data was collected for all study subjects, however, only those tests performed within 1 month of radiological examinations were included. All blood samples were processed using the standard protocol. Fasting venous blood samples in EDTA tubes were promptly centrifuged and stored at −20°C. Plasma Hcyt levels were determined using fluorescence polarization immunoassay with IMx (Abbott Laboratories, Abbott Park, Ill., USA). The inter-assay coefficient variation was between 3 and 6%.

### Assessment of Cerebral White Matter Hyperintensities

**Figure 1:**
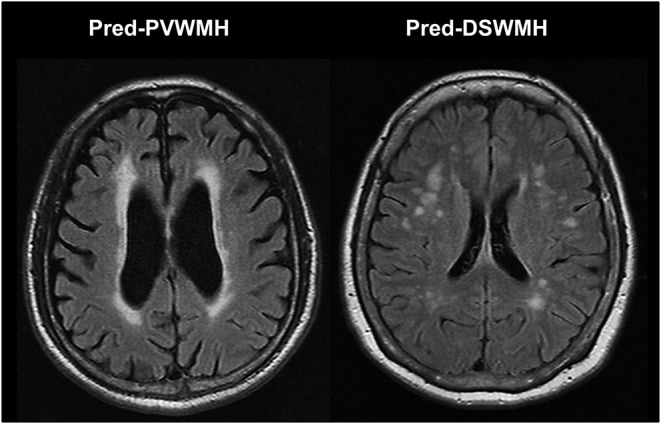
Illustrated case of the differentiation of predominant periventricular white matter hyperintensities (pred-PVWMH) and predominant deep subcortical white matter hyperintensities (pred-DSWMH).

The 1.5 T brain MR images were retrospectively examined in all study subjects. All series contained 16 axial images with a slice thickness of 7 mm with a 2 mm inter-slice gap. For segmentation and analysis of cerebral WMH, T1-weighted images (TR/TE = 560/14 ms) and FLAIR images (TR/TE = 9000/105 ms; inversion time, 2,500 ms) were used in accordance with the MR imaging protocol. The presence of cerebral WMH was evaluated on FLAIR images and the severity of cerebral WMH was assessed using the Fazekas et al. scoring system (19). The severity of the PVWMH was graded with the following scale: 0 = absent; 1 = cap- or pencil-thin lining; 2 = smooth halo; and 3 = irregular PVWMH extending into the deep white matter. The severity of the DSWMH was rated with the following scale: 0 = absent; 1 = punctuate foci; 2 = beginning of confluence of foci; and 3 = large confluent areas. The severity was graded as either none (0); mild (1); moderate (2); or a marked decrease in the attenuation of white matter (3). As mild WMH lesion is usually regarded as normal aging process, participants were divided into two groups according to their cerebral WMH scores: none to mild (cerebral WMH score 0–1) and moderate to severe (cerebral WMH score ≥2). The cerebral WMH predominance was defined as the difference of Fazekas score between PVWMH and DSWMH of two or more. For example, if one has a PVWMH score of three and a DSWMH score of one, then the case would be coded as predominantly PVWMH (pred-PVWMH). Another example is that if one has a PVWMH score of zero and a DSWMH score of two, then the case would be coded as predominantly DSWMH (pred-DSWMH). Figure 1 shows an example of the differentiation of pred-PVWMH and pred-DSWMH.

### Statistical Analysis

Continuous variables with a normal distribution are presented as mean ± SDs. Continuous data were examined using an unpaired two-sample *t*-test and categorical variables were compared using a chi-square test. Binary logistic regression was used to compare plasma Hcyt levels between those with cerebral WMH, PVWMH, DSWMH, pred-PVWMH, or pred-DSWMH and those without a corresponding lesion. Potential confounding factors were added to the analysis, including age, gender, hypertension, DM, hypercholesterolemia, smoking status, statin medication, CAOD, and eGFR. Odds ratios (ORs) and 95% confidence intervals (CIs) were calculated. Statistical analyses were conducted using SPSS (ver. 18.0, SPSS Inc, IL, USA). A two-sided *p* < 0.05 was considered statistically significant.

## Results

Demographic characteristics of study subjects are summarized in Table 1. Of the 937 subjects included in this study, when classified according to PVWMH, 677 (72.3%) subjects were in the none to mild group and 260 (27.7%) subjects were in the moderate to severe group. The moderate to severe PVWMH group had significantly higher plasma Hcyt levels compared to the none to mild group (*p* < 0.001). Age, HTN, DM, statin medication, and systolic BP were higher and eGFR was lower in the moderate to severe PVWMH group than in the none to mild group (Table 1). When classified according to DSWMH, 681 (72.7%) subjects were included in the none to mild group and 256 (27.3%) subjects were in the moderate to severe group. The moderate to severe DSWMH group had higher plasma Hcyt levels than those in the none to mild DSWMH group. Age, HTN, systolic BP, and diastolic BP were higher and eGFR was lower in the moderate to severe DSWMH group than in the none to mild group (Table 1).

**Table 1:** Clinical characteristics of subjects (*n* = 937) according to the location of cerebral WMH.

	**PVWMH**		***p***	**DSWMH**		***p***
	**None or mild (*n* = 677)**	**Moderate to severe (*n* = 260)**		**None or mild (*n* = 681)**	**Moderate to severe (*n* = 256)**	
Gender (male, %)	263 (38.8)	91 (35.0)	0.277	267 (39.2)	87 (34.0)	0.142
Age, year	64.8 ± 6.2	70.0 ± 5.8	<0.001	65.2 ± 6.4	68.9 ± 5.9	<0.001
Hypertension (%)	362 (53.5)	188 (72.3)	<0.001	372 (54.6)	178 (69.5)	<0.001
Diabetes mellitus (%)	138 (20.4)	82 (31.5)	<0.001	157 (23.1)	63 (24.6)	0.617
Hypercholesterolemia (%)	234 (34.6)	73 (28.1)	0.058	219 (32.2)	88 (34.4)	0.519
Current smoking status (%)	134 (19.8)	50 (19.2)	0.846	136 (20.0)	48 (18.8)	0.675
CAOD (%)	33 (4.9)	20 (7.7)	0.095	41 (6.0)	12 (4.7)	0.431
Statin medication (%)	175 (25.8)	47 (18.1)	0.012	169 (24.8)	53 (20.7)	0.187
SBP, mmHg	131.6 ± 17.1	135.9 ± 20.6	0.004	131.6 ± 17.5	136.0 ± 19.6	0.001
DBP, mmHg	80.0 ± 11.1	81.0 ± 12.4	0.212	79.8 ± 10.9	81.6 ± 12.7	0.037
Glucose, mg/dL	125.8 ± 48.3	128.7 ± 52.7	0.430	127.6 ± 48.3	124.1 ± 53.0	0.332
Total cholesterol, mg/dL	193.4 ± 39.7	192.4 ± 43.5	0.740	193.5 ± 40.2	192.2 ± 42.4	0.671
Triglyceride, mg/dL	150.5 ± 93.8	159.5 ± 113.26	0.214	153.7 ± 103.7	151.2 ± 88.1	0.734
eGFR, ml/min/1.73 m^2^	75.8 ± 16.3	68.3 ± 18.0	<0.001	75.0 ± 17.2	70.4 ± 16.4	<0.001
Hcyt, IU/L	10.4 ± 4.2	12.3 ± 5.9	<0.001	10.7 ± 4.3	11.6 ± 5.7	0.016

*CAOD, coronary arterial occlusive disease; SBP, systolic blood pressure; DBP, diastolic blood pressure; eGFR, estimated glomerular filtration rate; Hcyt, plasma total homocysteine; OR, odds ratio; CIs, confidence intervals*.

Linearity tests showed plasma Hcyt levels demonstrated a linear relationship between PVWMH scores (deviation from linearity, *F* = 0.501, *p* = 0.606) and DSWMH scores (deviation from linearity, *F* = 0.053, *p* = 0.948).

Results for the binary logistic regression analysis for the moderate to severe PVWMH and DSWMH groups are presented in Table 2. Plasma Hcyt was independently associated with the moderate to severe PVWMH group. Other variables independently associated with moderate to severe PVWMH were age, HTN, DM, low prevalence of statin medication, and low eGFR. Although plasma Hcyt levels were higher in the moderate to severe DSWMH group than in those with none to mild DSWMH in the univariate analysis, it was not statistically significant in the binary logistic regression analysis until adjusting for confounding factors. Other significant variables in the moderate to severe DSWMH group were age, HTN, hypercholesterolemia, and low prevalence of statin medication.

**Table 2:** Logistic regression analysis of subjects with moderate to severe PVWMH and DSWMH.

	**Moderate to severe PVWMH**	***p***	**Moderate to severe DSWMH**	***p***
	**OR (95% CIs)**		**OR (95% CIs)**	
Gender (male)	0.95 (0.65–1.38)	0.786	0.89 (0.62–1.27)	0.518
Age	1.13 (1.10–1.16)	<0.001	1.09 (1.07–1.12)	<0.001
Hypertension	1.78 (1.26–2.50)	0.001	1.59 (1.15–2.20)	0.005
Diabetes mellitus	1.61 (1.12–2.31)	0.009	0.97 (0.68–1.38)	0.849
Hypercholesterolemia	0.97 (0.61–1.55)	0.883	1.63 (1.05–2.53)	0.030
Current smoking status	1.15 (0.74–1.80)	0.541	1.09 (0.71–1.67)	0.692
CAOD	1.14 (0.60–2.16)	0.697	0.64 (0.32–1.28)	0.205
Statin medication	0.56 (0.33–0.96)	0.035	0.52 (0.32–0.86)	0.011
eGFR	0.63 (0.43–0.92)	0.017	0.99 (0.68–1.45)	0.961
Hcyt	1.06 (1.02–1.09)	0.002	1.02 (0.99–1.06)	0.150

*PVWMH, periventricular white matter hyperintensities; DSWMH, deep subcortical white matter hyperintensities; CAOD, coronary arterial occlusive disease; eGFR, estimated glomerular filtration rate; Hcyt, plasma total homocysteine; OR, odds ratio; CIs, confidence intervals*.

Next, we evaluate the prevalence of the moderate to severe PVWMH and DSWMH according to the quartile of plasma Hcyt level. Univariate analysis revealed that there were significant differences in age, gender, current smoking status, TG and eGFR across the Hcyt quartile groups (Supplementary Table 1). The prevalence of the moderate to severe PVWMH was significantly different across the Hcyt quartile groups. However, the prevalence of the moderate to severe DSWMH was not different across the Hcyt quartile groups. In logistic regression analysis, the prevalence of the moderate to severe PVWMH was independently associated with the highest Hcyt quartile group (Q4) compared to the lowest Hcyt quartile group (Q1) after adjusting vascular risk factors. On the other hand, the prevalence of the moderate to severe DSWMH was not different across the Hcyt quartile groups (Table 3).

**Table 3:** Logistic regression analysis of moderate to severe PVWMH and DSWMH based on plasma Hcyt quartiles.

	**Moderate to severe PVWMH**		**Moderate to severe DSWMH**	
	**OR (95% CIs)**	p^*^	**OR (95% CIs)**	p^*^
Q1 (<8.13 IU/L)	Ref	–	Ref	–
Q2 (8.13–9.99 IU/L)	0.62 (0.37–1.03)	0.067	1.14 (0.72–1.79)	0.582
Q3 (10.00–12.08 IU/L)	1.24 (0.76–2.02)	0.390	1.15 (0.96–0.98)	0.566
Q4 (>12.08 IU/L)	2.50 (1.44–4.34)	0.001	1.34 (0.79–2.28)	0.272

*Q1, Q2, Q3 and Q4 represent the quartile groups of plasma Hcyt level*.

* *Analyze after adjusting confounding factors (sex, age, hypertension, diabetes mellitus, hypercholesterolemia, current smoking status, coronary arterial occlusive disease, current statin medication, and estimated glomerular filtration rate)*.

*Ref represents the first quartile Hcyt group (Q1) as a reference group for analyses*.

*PVWMH, periventricular white matter hyperintensities; DSWMH, deep subcortical white matter hyperintensities; OR, odds ratio; CI, confidence interval*.

We assessed the relationship between plasma Hcyt levels and lesional predominance of cerebral WMH. A total of 114 subjects had predominant PVWMH lesions (Pred-PVWMH group) and 78 subjects had predominant DSWMH lesions (Pred-DSWMH group). A total of 351 subjects had no WMH lesions and the remaining 394 subjects had WMH without lesional predominance (both PVWMH and DSWMH group). Univariate analysis revealed significant differences in age, hypertension, diabetes, CAOD, SBP, eGFR, and plasma Hcyt levels across the four tested groups (Table 4). As represented in a 3-D bar graph of mean plasma Hcyt levels, according to PVWMH and DSWMH score (Supplementary Figure 1), the pred-PVWMH group trended toward increased mean plasma Hcyt levels compare to the pred-DSWMH group (Table 4 and Supplementary Figure 1). The mean Hcyt levels appeared to increase as the PVWMH score increased, irrespective of DSWMH scores, however, the DSWMH score did not show an increase irrespective of PWWMH score. Next, we conducted a multinomial logistic regression analysis to investigate the independent association of plasma Hcyt levels in the four tested groups (Table 5). The analysis demonstrated that increased age and plasma Hcyt levels were independently associated with the pred-PVWMH group. Increased age, male gender, a high prevalence of hypertension, having diabetes, and increased Plasma Hcyt levels were associated with the both PVWMH and DSWMH group. No significant variables were found in the pred-DSWMH group using a multinomial logistic regression analysis.

**Table 4:** Clinical characteristics of subjects (*n* = 937) according to the locational predominance of cerebral WMH.

	**No WMH (*n* = 351)**	**Pred-PVWMH (*n* = 114)**	**Pred-DSWMH (*n* = 78)**	**Both PVWMH and DSWMH (*n* = 394)**	***p***
Gender (male, %)	142 (40.5)	52 (45.6)	24 (30.8)	136 (34.5)	0.059
Age, year	63.2 ± 6.0	68.0 ± 5.9	64.7 ± 5.9	68.7 ± 5.9	<0.001
Hypertension (%)	174 (49.6)	57 (50.0)	46 (59.0)	273 (69.3)	<0.001
Diabetes mellitus (%)	65 (18.5)	30 (26.3)	14 (17.9)	111 (28.2)	0.009
Hypercholesterolemia (%)	116 (33.0)	34 (29.8)	25 (32.1)	132 (33.5)	0.902
Current smoking status (%)	72 (20.5)	23 (20.2)	12 (15.4)	77 (19.5)	0.780
CAOD (%)	19 (5.4)	12 (10.5)	0 (0.0)	22 (5.6)	0.020
Statin medication (%)	90 (25.6)	24 (21.1)	18 (23.1)	90 (22.8)	0.717
SBP, mmHg	130.9 ± 15.4	131.6 ± 18.6	130.6 ± 18.6	135.3 ± 20.1	0.006
DBP, mmHg	80.3 ± 10.9	78.9 ± 10.5	78.5 ± 11.1	81.0 ± 12.2	0.161
Glucose, mg/dL	124.8 ± 43.9	128.8 ± 47.3	128.9 ± 53.2	127.2 ± 54.2	0.819
Total cholesterol, mg/dL	194.2 ± 37.2	193.6 ± 48.7	199.3 ± 41.5	190.8 ± 41.2	0.350
Triglyceride, mg/dL	150.8 ± 101.6	142.0 ± 86.3	160.7 ± 97.7	156.6 ± 101.9	0.471
eGFR, ml/min/1.73 m^2^	77.3 ± 15.4	71.6 ± 19.7	73.3 ± 14.0	71.2 ± 17.8	<0.001
Hcyt, IU/L	10.0 ± 3.5	12.0 ± 6.1	9.6 ± 3.4	11.7 ± 5.3	<0.001

*pred-PVWMH, predominant periventricular white matter hyperintensities; pred-DSWMH, predominant deep subcortical white matter hyperintensities; CAOD, coronary arterial occlusive disease; SBP, systolic blood pressure; DBP, diastolic blood pressure; eGFR, estimated glomerular filtration rate; Hcyt, plasma total homocysteine*.

**Table 5:** Results of the multinomial logistic regression analysis of subjects with pred-PVWMH and pred-DSWMH.

	**No WMH**	**Pred-PVWMH**		**Pred-DSWMH**		**Both PVWMH and DSWMH**	
	**OR (95% CIs)**	**OR (95% CIs)**	p^*^	**OR (95% CIs)**	p^*^	**OR (95% CIs)**	p^*^
Age	Ref	1.12 (1.08–1.17)	<0.001	1.04 (1.00–1.08)	0.085	1.14 (1.11–1.17)	<0.001
Gender (male)	Ref	0.88 (0.52–1.48)	0.620	1.21 (0.64–2.27)	0.564	1.49 (1.00–1.20)	0.049
Hypertension	Ref	0.75 (0.48–1.18)	0.210	1.37 (0.82–2.30)	0.232	1.70 (1.22–2.37)	0.002
Diabetes mellitus	Ref	1.47 (0.87–2.49)	0.148	0.98 (0.51–1.89)	0.956	1.53 (1.04 0 2.24)	0.031
Hypercholesterolemia	Ref	1.10 (0.56–2.18)	0.781	0.90 (0.40–2.02)	0.798	1.20 (0.73–1.96)	0.468
Current smoking status	Ref	0.86 (0.47–1.57)	0.616	0.89 (0.42–1.89)	0.760	1.15 (0.73–1.78)	0.551
CAOD	Ref	1.40 (0.62–3.16)	0.424	NA	–	0.67 (0.33–1.36)	0.271
Statin medication	Ref	0.68 (0.32–1.45)	0.323	0.93 (0.38–2.25)	0.863	0.66 (0.39–1.13)	0.131
eGFR	Ref	0.99 (0.98–1.01)	0.188	0.99 (0.97–1.00)	0.082	1.00 (0.98–1.01)	0.304
Hcyt	Ref	1.08 (1.03–1.14)	0.005	0.95 (0.87–1.04)	0.274	1.08 (1.03–1.13)	0.001

* *Analysis after adjusting for confounding factors (sex, age, hypertension, diabetes mellitus, hypercholesterolemia, current smoking status, CAOD, current statin medication, estimated glomerular filtration rate, and plasma total homocysteine level)*.

*No WHH group was regarded as reference (Ref) for analysis. NA means not available because there was no case of CAOD in the pred-DSWMH group*.

*CAOD, coronary arterial occlusive disease; Hcyt, plasma total homocysteine; OR, odds ratio; CIs, confidence intervals*.

## Discussion

The current study demonstrated that elevated plasma Hcyt levels were independently associated with cerebral WMH. Specifically, a multivariate logistic regression analysis demonstrated that plasma Hcyt levels were associated with PVWMH. These results suggest the possibility that dysregulated Hcyt metabolism plays a role in the pathogenesis of cerebral WMH at the periventricular region in the brain.

Elevated plasma Hcyt induces endothelial dysfunction and extracellular matrix proliferation, leading to vascular damage. Several studies have suggested that elevated plasma Hcyt levels are associated with an increased risk of various vascular diseases, including cerebral WMH (15–17, 26). A Rotterdam Scan Study found that the overall risk of having severe cerebral WMH was strongly associated with plasma Hcyt levels (20). Plasma Hcyt levels were also significantly associated with an increased risk of cerebral WMH progression after adjusting for other confounding factors (27). Another recent study also suggested that plasma Hcyt levels were correlated with cerebral WMH volume in a dose-dependent manner (28). Of note, however, most previous studies have specifically focused on the association of plasma Hcyt levels with overall WMH burden.

Because of a paucity of data, it is controversial whether plasma Hcyt levels have a discriminative effect between PVWMH and DSWMH. Some studies have shown that plasma Hcyt levels were primarily related to PVWMH as opposed to DSWMH, whereas the others have shown the opposite result. Gao et al. found that plasma Hcyt levels were not only related to overall WMH burden but also to WMH locations, which are distributed within the periventricular and frontal areas in patients with acute ischemic stroke (24). In addition, a population-based autopsy study demonstrated that plasma Hcyt levels were associated with PVWMH but not DSWMH using postmortem MRIs (25). Contrarily, other studies have shown that plasma Hcyt levels were related to an increase in DSWMH in a healthy community sample, but the association was only found in men (29). Another study suggested that plasma Hcyt levels were associated with DSWMH in patients with Alzheimer's dementia (15). The inconsistency of these results may be due to different clinical characteristics of patients, the definition of PVWMH and DSWMH lesions, and the rating scaling method of WMH across studies. Past studies have primarily investigated the relationship between plasma Hcyt levels and WMH in neurologically-ill patients, such as those with acute stroke or Alzheimer disease, which may affect Hcyt metabolism and WMH development (15, 24, 25). The current study demonstrated that elevated plasma Hcyt levels were associated with PVWMH but not with DSWMH in healthy subjects. This finding provides strong evidence of a crucial association between plasma Hcyt levels and PVWMH without any neurological disease-related confounders.

It is generally well-known that PVWMH and DSWMH lesions occur concurrently. As age increases, the WMH lesions typically develop in one white matter region and then spread toward another white matter region (from the periventricular region to the deep subcortical region, or *vice versa*). In the current study, 67% of cases with cerebral WMH showed both PVWMH and DSWMH without lesional predominance. Additionally, the confidence intervals for DSWMH and PVWMH in the multivariable analysis are substantially overlapping (Table 2). It is difficult to reach firm conclusions from these finding, which may have contributed to the conflicting results across studies. In the present study, we particularly focused on cases with lesional predominance of cerebral WMH (pred-PVWMH and pred-DSWMH groups) to gain solid evidence of the impact of Hcyt on the predominant development of PVWMH lesions. In accordance with previous studies (8, 9, 20), multinomial analyses revealed that cases with cerebral WMH without lesional predominance (both PVWMH and DSWMH group) were old and coexisted with a high prevalence of hypertension, diabetes, and plasma Hcyt levels. However, only age and plasma Hcyt levels were significantly associated in those with pred-PVWMH lesions. Conversely, plasma Hcyt levels were not associated with the development of DSWMH. Based on these findings, we suggest that plasma Hcyt is a crucial culprit for the development of cerebral WMH at the periventricular region, but not at the deep subcortical region.

While the current study suggests a differential impact of Hcyt on PVWHM and DSWMH, it is not possible to fully explain the mechanisms by which this occurs, however, there are some plausible explanations. One proposed theory is that PVWMH and DSWMH reflect different histopathological and etiological features (1, 19–23, 30–32). Postmortem studies have shown that PVWMH has discontinuous ependymal loss (resulting in high extra cellular fluid content), sub-ependymal gliosis, a loosening of the white matter fibers, and loss of myelin, which are non-ischemic in nature. The DSWMH shows more variable axonal loss, multiple small vacuolations, and increased tissue loss in more severe lesions, suggesting possible arteriosclerosis and microcystic infarction in addition to demyelination and gliosis (23, 31, 33). Smooth PVWMH, including caps and halos, may be linked to non-ischemic tissue damage, the increase of interstitial cerebrospinal fluid (CSF) leakage, while irregular PVWMH are more likely determined by chronic hypoperfusion. Contrarily, DSWMH may be more likely to be ischemic in nature (31). Previous pathology studies have also suggested that DSWMH may present more hypoxic/ischemic damage whereas PVWMH may have a greater inflammatory/metabolic component (34, 35). Because of the anatomical differences between the arterioles at the basal ganglia (near the periventricular region) and superficial perforating arterioles at the deep subcortical region, tissue around the basal ganglia arterioles is more protected from the effects of vascular disease than is tissue around the superficial perforating arterioles (23, 31, 33, 34). These differences, therefore, support an association between plasma Hcyt and PVWMH. Hcyt inhibits endothelial nitric oxide via the production of reactive oxygen species or the accumulation of asymmetric dimethylarginine, which would, in turn, lead to functional suppression of the blood-brain barrier (BBB) (36, 37). Hcyt destroys endothelium, either functionally or mechanically, through an inflammatory cascade and a BBB disruption could lead to a perivascular infiltration of toxic materials into neural tissues or the blockage of interstitial fluid clearance via the glymphatic pathway (38–40). Consequently, the cause of these differences may be a direct effect of the subsequent loss of axons in fiber tracts, which runs near the lateral ventricles, or it may be an indirect effect of subsequent ventricular dilatation and ruptures of the ependymal lining, with increased leakage of CSF into the surrounding ventricles (24, 25, 28). These findings provide plausible explanations of why Hcyt is more associated with PVWMH than with DSWMH.

The present study has several limitations. First, this is a single-center study and the sample size was too small to be generalized. Therefore, a large-scaled external validation of the current study is necessary. Second, cerebral WMH are complex diseases with multiple causal factors. We did not investigate possible candidates for risk factors of cerebral WMH such as C-reactive protein (CRP), interleukin-6 (IL-6), vitamin B12, or apolipoprotein E 4 allele (41–43). Third, this was a retrospective study, which may have selection or confounding bias. Fourth, we did not measure plasma Hcyt levels and conduct the brain MRI serially, thus preventing us from concluding a causal relationship between plasma Hcyt and cerebral WMH. Fifth, the predominance assignments using the difference of Fazekas score between two regions ≥2 were first shown in our study. Basically, our study was based on the idea that the pathophysiological mechanism of PVWMH and DSWMH is different. Therefore, it was considered that giving the predominance is the best way without using MRI volumetry. However, MRI volumetry should be used to provide more accurate and reliable predominance, so a large scale study using MRI volumetry will be needed in the future. Finally, we cannot exclude the possibility that high levels of plasma Hcyt is an epiphenomenon of other pathologic conditions, such as systemic infection or an inflammatory state. However, this possibility is remote, as only asymptomatic healthy individuals were included in the study and individuals with a suspicious clinical infection or fever were excluded.

## Conclusions

Results from our study revealed that elevated plasma Hcyt yielded a strong pathological impact on the development of WMH at the periventricular region in a neurologically healthy population. Our study reassuringly indicates that different pathogeneses exist for PVWMH and DSWMH and that dysregulated Hcyt metabolism plays a role in the development of PVWMH.

## Data Availability Statement

All datasets generated for this study are included in the article/Supplementary Material.

## Ethics Statement

The studies involving human participants were reviewed and approved by Institutional Ethical Committee of the CHA Bundang Medical Center. The patients/participants provided their written informed consent to participate in this study.

## Author Contributions

KL: conception, design, drafting of manuscript, acquisition of data, and final approval of manuscript. M-HW, DC, and J-WC: design, acquisition of data, revision of manuscript, and final approval of manuscript. N-KK: design, acquisition of data, interpretation of data, revision of manuscript, and final approval of manuscript. O-JK: concept, design, acquisition of data, revision of manuscript, and final approval of manuscript. S-HO: conception, design, analysis and interpretation of data, drafting of manuscript, revision of manuscript, and final approval of manuscript.

### Conflict of Interest

The authors declare that the research was conducted in the absence of any commercial or financial relationships that could be construed as a potential conflict of interest.
