# Supplementary material for: Differential Impact of Plasma Homocysteine Levels on the Periventricular and Subcortical White Matter Hyperintensities on the Brain
Source: Front Neurol. 2019 Nov 7;10:1174. doi: 10.3389/fneur.2019.01174 (PMC6856638; doi:10.3389/fneur.2019.01174)
Supplement: Supplementary file 1 [file Table_1.docx]

**Supplementary materials**

Supplementary Table 1. Clinical characteristics of subjects based on plasma Hcyt quartile

|  | Q1 (< 8.13 IU/L)  0  (  (n=234) | Q2 (8.13-9.99 IU/L)  (n=234) | Q3 (10.00-12.08 IU/L)  (n=235) | Q4 (> 12.08 IU/L)  (n=234) | p |
| --- | --- | --- | --- | --- | --- |
| Gender (male, %) | 48 (20.5) | 61 (26.1) | 103 (43.8) | 142 (60.7) | <0.001 |
| Age, years | 64.8 ± 6.4 | 65.8 ± 6.6 | 66.5 ± 6.3 | 67.8 ± 6.3 | <0.001 |
| Hypertension (%) | 123 (52.6) | 139 (59.4) | 143 (60.9) | 145 (62.0) | 0.160 |
| Diabetes mellitus (%) | 55 (23.5) | 44 (18.8) | 56 (23.8) | 65 (27.8) | 0.153 |
| Hypercholesterolemia (%) | 76 (32.5) | 76 (32.5) | 80 (34.0) | 75 (32.1) | 0.970 |
| Current smoking status (%) | 32 (13.7) | 29 (12.4) | 52 (22.1) | 71 (30.3) | <0.001 |
| CAOD (%) | 10 (4.3) | 15 (6.4) | 11 (4.7) | 17 (7.3) | 0.450 |
| Statin medication (%) | 60 (25.6) | 55 (23.5) | 54 (23.0) | 53 (22.6) | 0.872 |
| SBP, mmHg | 132.3 ± 18.0 | 131.8 ± 15.7 | 133.1 ± 18.1 | 134.1 ± 20.9 | 0.525 |
| DBP, mmHg | 79.8 ± 12.6 | 80.2 ± 10.9 | 81.0 ± 10.8 | 80.2 ± 11.4 | 0.735 |
| Glucose, mg/dL | 128.5 ± 56.3 | 122.8 ± 48.1 | 127.8 ± 46.0 | 127.5 ± 47.5 | 0.581 |
| Total cholesterol, mg/dL | 195.0 ± 40.4 | 192.5 ± 40.2 | 195.3 ± 40.7 | 189.8 ± 41.9 | 0.434 |
| Triglyceride, mg/dL | 141.2 ± 87.1 | 147.1 ± 87.7 | 157.9 ± 93.5 | 165.9 ± 124.4 | 0.036 |
| eGFR, mL/min/1.73 m^2^ | 79.3 ± 14.8 | 74.7 ± 16.2 | 73.9 ± 15.5 | 67.1 ± 19.3 | <0.001 |
| Moderate to severe PVWMH (%) | 50 (21.4) | 43 (18.4) | 69 (29.4) | 98 (41.9) | <0.001 |
| Moderate to severe DSWMH (%) | 52 (22.2) | 64 (27.4) | 68 (25.1) | 72 (30.8) | 0.189 |

Q1, Q2, Q3, and Q4 represent the quartile groups of plasma Hcyt level. Values are presented as percentages or means ± standard deviation. P-values were derived from an analysis of variance (ANOVA) for continuous variables and chi-square test for categorical variables. CAOD: coronary arterial occlusive disease, SBP: systolic blood pressure, DBP: diastolic blood pressure, eGFR: estimated glomerular filtration rate, PVWMH: periventricular white matter hyperintensities, DSWMH: deep subcortical white matter hyperintensities

Supplementary Figure 1.

3-D bar graph of Mean plasma Hcyt level according to PVWMH and DSWMH scores

**
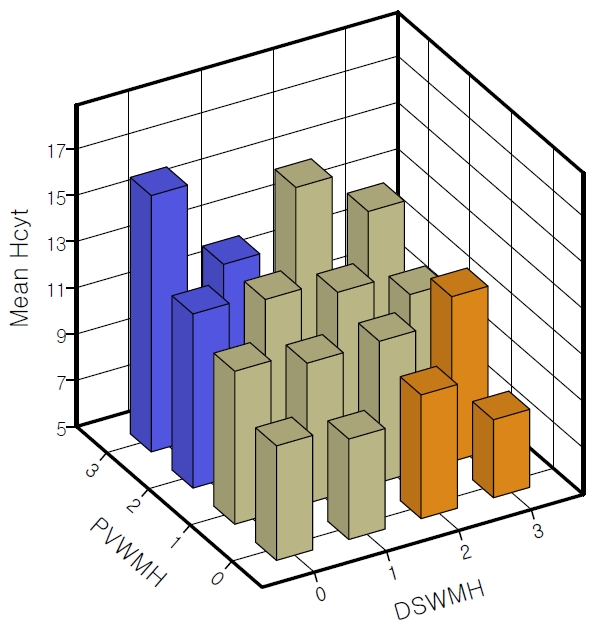
**

Blue-colored bars indicate the mean plasma Hcyt levels of the pred-PVWMH group and orange-colored bars indicate the mean plasma Hcyt levels of the pred-DSWMH group.

Abbreviation: PVWMH: periventricular white matter hyperintensities, DSWMH: deep subcortical white matter hyperintensities, Hcyt: homocysteine
